# Proteomic analysis of the defense response to *Magnaporthe oryzae* in rice harboring the blast resistance gene *Piz-t*

**DOI:** 10.1186/s12284-018-0240-3

**Published:** 2018-08-15

**Authors:** Dagang Tian, Liu Yang, Zaijie Chen, Ziqiang Chen, Feng Wang, Yuanchang Zhou, Yuming Luo, Liming Yang, Songbiao Chen

**Affiliations:** 1 Biotechnology Research Institute, Fujian Key Laboratory of Genetic Engineering for Agriculture, Fujian Academy of Agricultural Sciences, Fuzhou, 350003 China; 2 College of Biology and the Environment, Nanjing Forestry University, Nanjing, 210037 China; 3 Institute of Oceanography, Marine Biotechnology Center, Minjiang University, Fuzhou, 350108 China; 4 College of Crop Science, Fujian Agricultural and Forestry University, Fuzhou, 350002 China; 5 College of Life Sciences, Huaiyin Normal University, Huaian, 223300 China

**Keywords:** Proteomic analysis, Rice blast disease, Resistance gene, *Piz-t*, iTRAQ

## Abstract

**Background:**

Rice blast (caused by *Magnaporthe oryzae*) is one of the most destructive diseases of rice. While many blast resistance (*R*) genes have been identified and deployed in rice cultivars, little is known about the *R* gene-mediated defense mechanism. We used a rice transgenic line harboring the resistance gene *Piz-t* to investigate the *R* gene-mediated resistance response to infection.

**Results:**

We conducted comparative proteome profiling of the *Piz-t* transgenic Nipponbare line (NPB-Piz-t) and wild-type Nipponbare (NPB) inoculated with *M. oryzae* at 24, 48, 72 h post-inoculation (hpi) using isobaric tags for relative and absolute quantification (iTRAQ) analysis. Comparative analysis of the response of NPB-Piz-t to the avirulent isolate KJ201 and the virulent isolate RB22 identified 114 differentially expressed proteins (DEPs) between KJ201-inoculated NPB-Piz-t (KJ201-Piz-t) and mock-treated NPB-Piz-t (Mock-Piz-t), and 118 DEPs between RB22-inoculated NPB-Piz-t (RB22-Piz-t) and Mock-Piz-t. Among the DEPs, 56 occurred commonly in comparisons KJ201-Piz-t/Mock-Piz-t and RB22-Piz-t/Mock-Piz-t. In a comparison of the responses of NPB and NPB-Piz-t to isolate KJ201, 93 DEPs between KJ201-Piz-t and KJ201-NPB were identified. DEPs in comparisons KJ201-Piz-t/Mock-Piz-t, RB22-Piz-t/Mock-Piz-t and KJ201-Piz-t/KJ201-NPB contained a number of proteins that may be involved in rice response to pathogens, including pathogenesis-related (PR) proteins, hormonal regulation-related proteins, defense and stress response-related proteins, receptor-like kinase, and cytochrome P450. Comparative analysis further identified 7 common DEPs between the comparisons KJ201-Piz-t/KJ201-NPB and KJ201-Piz-t/RB22-Piz-t, including alcohol dehydrogenase I, receptor-like protein kinase, endochitinase, similar to rubisco large subunit, NADP-dependent malic enzyme, and two hypothetical proteins.

**Conclusions:**

Our results provide a valuable resource for discovery of complex protein networks involved in the resistance response of rice to blast fungus.

**Electronic supplementary material:**

The online version of this article (10.1186/s12284-018-0240-3) contains supplementary material, which is available to authorized users.

## Background

Rice (*Oryza sativa* L.) is one of the most important food crops worldwide and is a major source of calories for half the world’s population. Rice blast, caused by the ascomycete fungus *Magnaporthe oryzae*, is one of the most damaging diseases in rice. The blast fungus infects rice leaves, stems, nodes, panicles, and roots at all developmental stages (Wilson and Talbot 2009), causing significant yield losses in rice production (Dean et al. 2005; Ebbole 2007). The use of resistant cultivars is the most effective, economical, and environmentally friendly way of controlling rice blast (Tian et al. 2016). In recent decades, over 100 resistance (*R*) genes have been identified and at least 25 have been cloned (Singh et al. 2015), providing an extensive genetic resource for breeding blast-resistant rice. However, little is known about the *R* gene-mediated defense mechanism in rice.

Because of the high degree of pathogenic variability of *M. oryzae* (Dean et al. 2005), *R* gene-mediated resistance is prone to breakdown, making it difficult to breed durable blast resistance in rice. A better understanding of the *R* gene-mediated host defense mechanism may improve breeding for durable blast resistance. Over the past decade, high-throughput gene and protein profiling techniques have enabled investigation of the molecular processes involved in the defense responses of rice to *M. oryzae* infection (Li et al. 2006; Vergne et al. 2007, 2010; Bagnaresi et al. 2012; Gupta et al. 2012; Kim et al. 2013; Li et al. 2014, 2015; Zhang et al. 2016; Jain et al. 2017). These large-scale transcriptomic and proteomic analyses have identified rice genes and/or proteins that were differentially expressed upon infection by *M. oryzae* and provided overviews of biological processes characterizing rice-*M. oryzae* interactions.

The rice blast *R* gene *Piz-t*, which encodes a nucleotide-binding site-leucine-rich repeat protein, is a member of the *Pi2/9* multi-allelic gene family (Zhou et al. 2006). *Piz-t* corresponds in a gene-for-gene fashion to the *M. oryzae* avirulence (*Avr*) gene *AvrPiz-t* (Li et al. 2009). AvrPiz-t is a small, secreted protein that is translocated into the host cytoplasm. Recent studies have revealed that AvrPiz-t targets two rice ubiquitin E3 ligases, APIP6 (AvrPiz-t Interacting Protein 6) and APIP10, to suppress PAMP-triggered immunity in rice (Park et al. 2012, 2016). AvrPiz-t also interacts with rice’s bZIP-type transcription factor APIP5 (Wang et al. 2016), and its Nup98 family protein APIP12 (Tang et al. 2017), and K^+^ channel protein OsAKT1 APIP7 (Shi et al. 2018) to suppress host basal defense. Conversely, Piz-t recognizes the AvrPiz-t signal through APIP5 and APIP10, and activates effector-triggered immunity in rice (Park et al. 2016; Wang et al. 2016). While the findings revealed aspects of the functions of AvrPiz-t/APIPs/Piz-t, the mechanisms underlying Piz-t-mediated rice resistance to blast fungus remain largely unknown.

In this study, we performed isobaric tags for relative and absolute quantification (iTRAQ)-based proteomics analysis of rice in response to *M. oryzae* infection. We compared the protein expression profiles of Nipponbare and a transgenic Nipponbare line harboring the *Piz-t* gene inoculated with virulent and avirulent isolates at 24, 48, 72 h post-inoculation (hpi), respectively. A number of differentially expressed proteins (DEPs) that may be involved in rice response to pathogens were identified, including pathogenesis-related (PR) proteins, hormonal regulation-related proteins, defense and stress response-related proteins, receptor-like kinase, and cytochrome P450. We expected that our results would provide a valuable resource for the discovery of complex protein networks involved in the resistance response of rice to blast fungus.

## Results

### Disease assessment and iTRAQ analysis

**Fig. 1:**
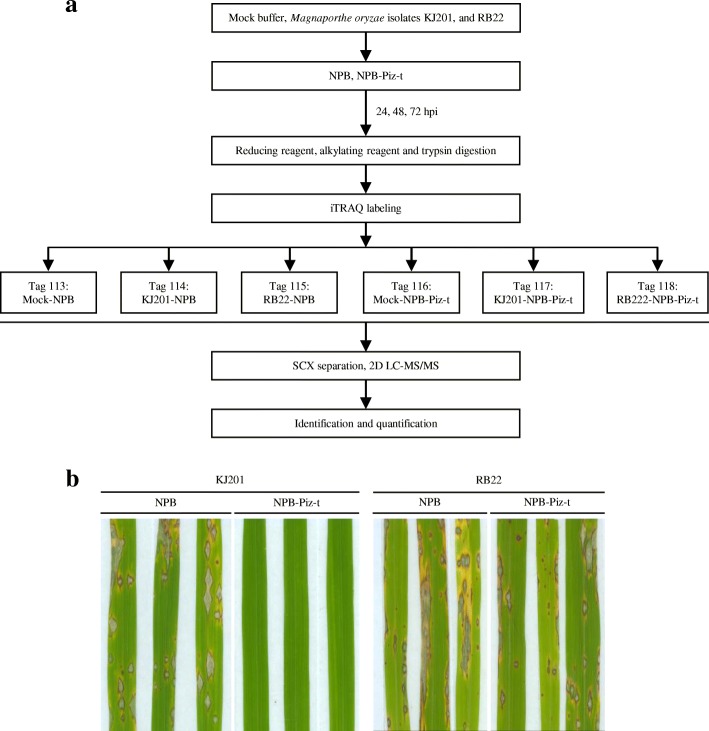
Schematic representation of the experimental design. **a** The workflow for proteomic analysis of the defense response to *M. oryzae* in rice harboring the blast resistance *Piz-t* gene using iTRAQ 2D LC-MS/MS technology. **b** Phenotypes of the wild-type rice cultivar Nipponbare (NPB) and transgenic Nipponbare line harboring the *Piz-t* gene (NPB-Piz-t) inoculated with *M. oryzae* isolates KJ201 and RB22, respectively

To compare the responses of wild-type Nipponbare (NPB) and Nipponbare harboring the *Piz-t* gene (NPB-Piz-t) to infection with avirulent and virulent *M. oryzae* isolates, we inoculated NPB and NPB-Piz-t with isolates KJ201 and RB22, respectively. An outline of the experimental design is shown in Fig. 1a. At 24, 48, and 72 hpi, no obvious symptoms of infection were observed on all tested rice seedlings. At 7 days post inoculation, while KJ201-inoculated NPB-Piz-t rice seedlings showed no disease symptoms, severe blast lesions were observed in NPB seedlings inoculated with KJ201 and RB22, and in NPB-Piz-t seedlings inoculated with RB22 (Fig. 1b), indicating the success of the inoculation experiment.

Proteins extracted from leaves of NPB and NPB-Piz-t inoculated with *M. oryzae* isolates were subjected to iTRAQ analysis. After data filtering to eliminate low-scoring spectra (false discovery rate (FDR) < 0.01), 2743, 1991, and 1839 proteins were identified from both mock-treated and *M. oryzae*-inoculated rice seedlings at 24, 48, and 72 hpi, respectively (Table 1). DEPs between *M. oryzae*-inoculated and mock-treated leaves were identified according to the criteria of fold change ≥1.5 or ≤ 0.67 and *P* < 0.05. In the NPB background, 85 proteins were identified as DEPs between the KJ201-inoculated sample (referred to as KJ201-NPB) and the mock-treated sample (referred to as Mock-NPB), including 11, 30, and 47 proteins at 24, 48, and 72 hpi, respectively; 120 proteins were identified as DEPs between the RB22-inoculated sample (referred to as RB22-NPB) and Mock-NPB, including 17, 41, and 67 proteins at 24, 48, and 72 hpi, respectively (Table 1, Additional file 1: Table S1). In the NPB-Piz-t background, 114 proteins were identified as DEPs between the KJ201-inoculated sample (referred to as KJ201-Piz-t) and the mock-treated sample (referred to as Mock-Piz-t), including 23, 43, and 50 proteins at 24, 48, and 72 hpi, respectively; 118 proteins were identified as DEPs between the RB22-inoculated sample (referred to as RB22-Piz-t) and Mock-Piz-t, including 17, 37, and 65 proteins at 24, 48, and 72 hpi, respectively (Table 1, Additional file 1: Table S1).

**Table 1:** Distribution of spectra, peptide and protein identified at 24, 48 and 72 hpi

hpi	Spectra	Peptides	Proteins	Differential expressed proteins					
				KJ201-NPB/Mock-NPB	RB22-NPB/Mock-NPB	KJ201-Piz-t/Mock-Piz-t	RB22-Piz-t/Mock-Pizt	KJ201-Piz-t/KJ201-NPB	KJ201-Piz-t/RB22-Pizt
24 h	27133	16411	2743	11 (10↑, 1↓)	17 (4↑, 13↓)	23 (10↑, 13↓)	17 (8↑, 9↓)	40 (18↑, 22↓)	9 (7↑, 2↓)
48 h	27969	12125	1991	30 (21↑, 9↓)	41 (20↑, 21↓)	43 (35↑, 8↓)	37 (30↑,7↓)	32 (27↑, 5↓)	20 (8↑, 12↓)
72 h	29096	12593	1839	47 (29↑, 18↓)	67 (21↑,46↓)	50 (42↑, 8↓)	65 (50↑, 15↓)	24 (15↑, 9↓)	24 (7↑,17↓)
Total	ND	ND	ND	85 (55↑, 27↓, 2↑↑, 1↑↓)	120 (42↑, 73↓, 1↑↑, 1↑↓, 3↓↓)	114 (83↑, 29↓, 2↑↑)	118 (87↑, 30↓, 1↑↓)	93 (57↑, 33↓, 1↑↑, 1↑↓, 1↓↓)	53 (22↑, 31↓)

↑ upregulated; ↓ downregulated; ↑↑ upregulated at two different hpi; ↓↓ downregulated at two different hpi; ↑↓ upregulated and downregulated at two different hpi; respectively; *ND* not determined

### Validation of differentially expressed proteins by qRT-PCR and western blotting

**Fig. 2:**
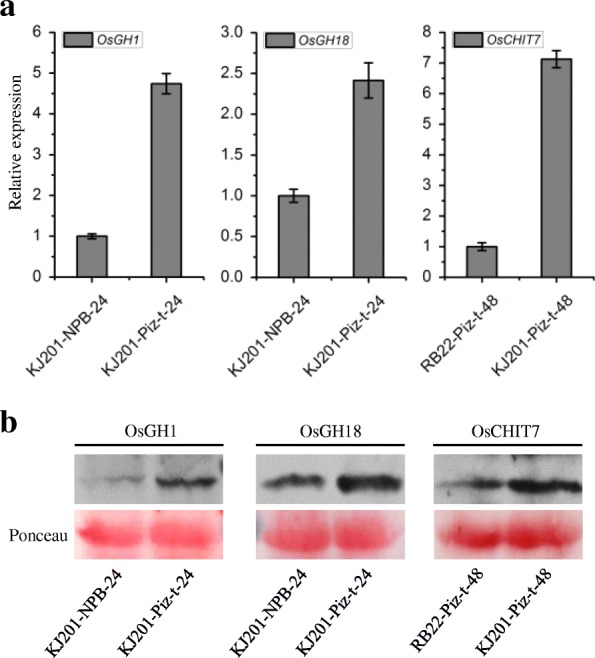
qRT-PCR (**a**) and western blot (**b**) confirmation of the three differentially expressed proteins identified by iTRAQ

Quantitative reverse-transcription PCR (qRT-PCR) and western blotting were used to validate the expression patterns of three proteins identified by iTRAQ. qRT-PCR results showed that the transcription level of OsGH1 (gi|218199777) in KJ201-Piz-t at 24 hpi was approximately 4.7 times that in KJ201-NPB at 24 hpi (Fig. 2a). Western blot results showed that the accumulation of OsGH1 in KJ201-Piz-t at 24 hpi was obviously stronger than that in KJ201-NPB at 24 hpi (Fig. 2b). These results correlated with the iTRAQ data. Similarly, the results of qRT-PCR and western blotting revealed that the expression patterns of OsGH18 (gi|55168113) and OsCHIT7 (gi|20196) were consistent with the iTRAQ results (Fig. 2).

### Differential proteomic profiles in NPB-Piz-t in response to virulent and avirulent *M. oryzae* isolates

**Fig. 3:**
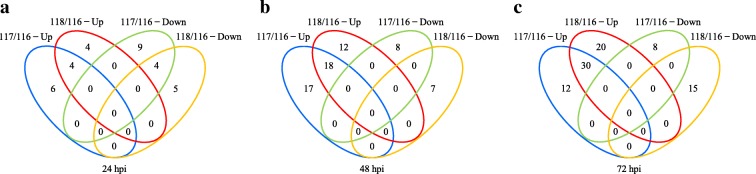
Venn diagrams of DEPs in NPB-*Piz-t* inoculated with *M. oryzae* isolates at 24 (**a**), 48 (**b**), and 72 hpi (**c**), respectively. 116: mock-treated NPB-Piz-t; 117: KJ201-inoculated NPB-Piz-t; 118: RB22-inoculated NPB-Piz-t; Up: upregulated proteins; Down: Downregulated proteins

We analyzed the proteomic profiles of NPB-Piz-t in response to the avirulent isolate KJ201 and the virulent isolate RB22. Compared with Mock-Piz-t, 10, 35, and 42 proteins were identified to be differentially upregulated in KJ201-Piz-t at 24, 48, and 72 hpi, and 13, 8, and 8 proteins were differentially downregulated in KJ201-Piz-t at 24, 48 and 72 hpi, respectively (Table 1). In RB22-Piz-t, 8, 30, and 50 proteins were differentially upregulated at 24, 48 and 72 hpi, and 9, 7 and 15 proteins were differentially downregulated at 24, 48 and 72 hpi, respectively (Table 1). In total, 85 proteins were differentially upregulated, and 29 proteins were downregulated in KJ201-Piz-t, and 87 and 30 proteins were differentially upregulated or downregulated, respectively, in BR22-Piz-t (Table 1). Among the upregulated proteins, 4, 18, and 30 were differentially upregulated in both KJ201-Piz-t and RB22-Piz-t at 24, 48, and 72 hpi, respectively (Fig. 3a-c). While 4 proteins were differentially downregulated in both KJ201-Piz-t and RB22-Piz-t at 24 hpi (Fig. 3a), no proteins were shared between KJ201-Piz-t and RB22-Piz-t at 48 or 72 hpi (Fig. 3b, c).

**Fig. 4:**
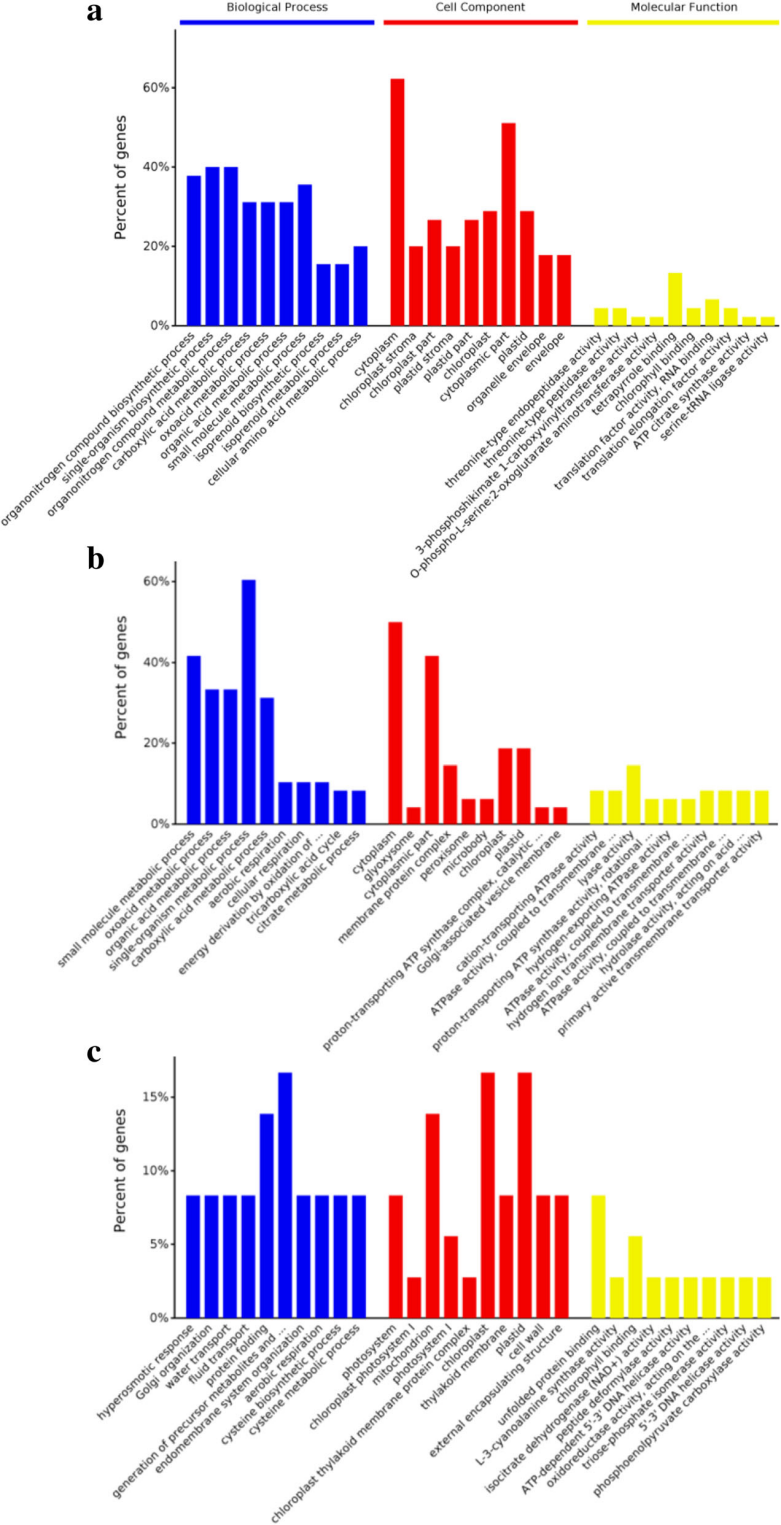
GO analysis of DEPs in NPB-Piz-t in response to *M. oryzae* inoculation. **a** GO analysis of DEPs specific to comparison KJ201-Piz-t/Mock-Piz-t. **b** GO analysis of DEPs specific to comparison RB22-Piz-t/Mock-Piz-t. **c** GO analysis of common DEPs in comparisons KJ201-Piz-t/Mock-Piz-t and RB22-Piz-t/Mock-Piz-t. The results are summarized in three main categories: biological process, cellular component, and molecular function

Using Gene Ontology (GO) analysis, DEPs were classified into biological process, cellular component, and molecular function terms (Fig. 4, Additional file 2: Table S2). Analysis of DEPs specific to comparison KJ201-Piz-t/Mock-Piz-t showed that the most common biological process categories were associated with metabolic and biosynthetic processes; the most common cellular component categories with cytoplasm and cytoplasmic parts; and the most common molecular function categories with binding and catalytic activity (Fig. 4a, Additional file 2: Table S2). In the case of DEPs specific to comparison RB22-Piz-t/Mock-Piz-t, the most common biological process categories were associated with metabolic process; the most common cellular component categories were cytoplasm and cytoplasmic parts; and the most common molecular function category was catalytic activity (Fig. 4b, Additional file 2: Table S2). For common DEPs in comparisons KJ201-Piz-t/Mock-Piz-t and RB22-Piz-t/Mock-Piz-t, the most common biological process categories were associated with the generation of precursor metabolites and protein folding; the most common cellular component categories were chloroplast, plastid, and mitochondrion; and the most common molecular function categories were catalytic activity and binding (Fig. 4c, Additional file 2: Table S2).

**Fig. 5:**
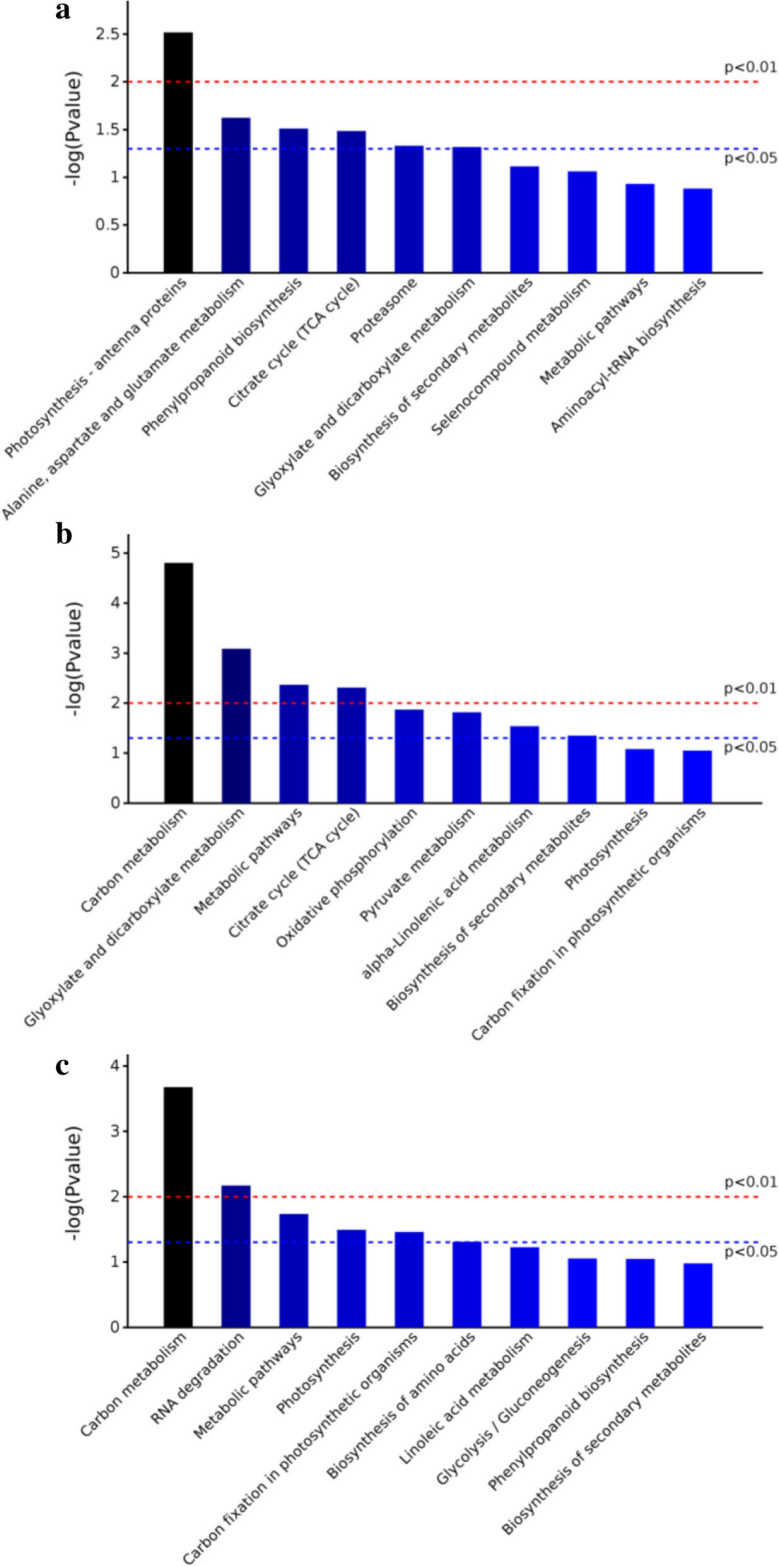
KEGG classification of DEPs in NPB-Piz-t in response to *M. oryzae* inoculation. **a** KEGG classification of DEPs specific to comparison KJ201-Piz-t/Mock-Piz-t. **b** KEGG classification of DEPs specific to comparison RB22-Piz-t/Mock-Piz-t. **c** KEGG classification of common DEPs in comparisons KJ201-Piz-t/Mock-Piz-t and RB22-Piz-t/Mock-Piz-t

Kyoto Encyclopedia of Genes and Genomes (KEGG) analysis revealed that DEPs specific to comparison KJ201-Piz-t/Mock-Piz-t were mainly involved in pathways related to photosynthetic antenna proteins; alanine, aspartate, and glutamate metabolism; phenylpropanoid biosynthesis; citrate (TCA) cycle; proteasome; and glyoxylate and dicarboxylate metabolism (Fig. 5a). DEPs specific to comparison RB22-Piz-t/Mock-Piz-t were mainly associated with the metabolism of carbon, glyoxylate and dicarboxylate; pyruvate and alpha-linolenic acid; and pathways related to metabolic, TCA cycle, oxidative phosphorylation, and biosynthesis of secondary metabolites (Fig. 5b). The most highly enriched pathways in common DEPs in comparisons KJ201-Piz-t/Mock-Piz-t and RB22-Piz-t/Mock-Piz-t were associated with carbon metabolism, RNA degradation, metabolic pathways, photosynthesis, carbon fixation in photosynthetic organisms, and biosynthesis of amino acids (Fig. 5c).

**Fig. 6:**
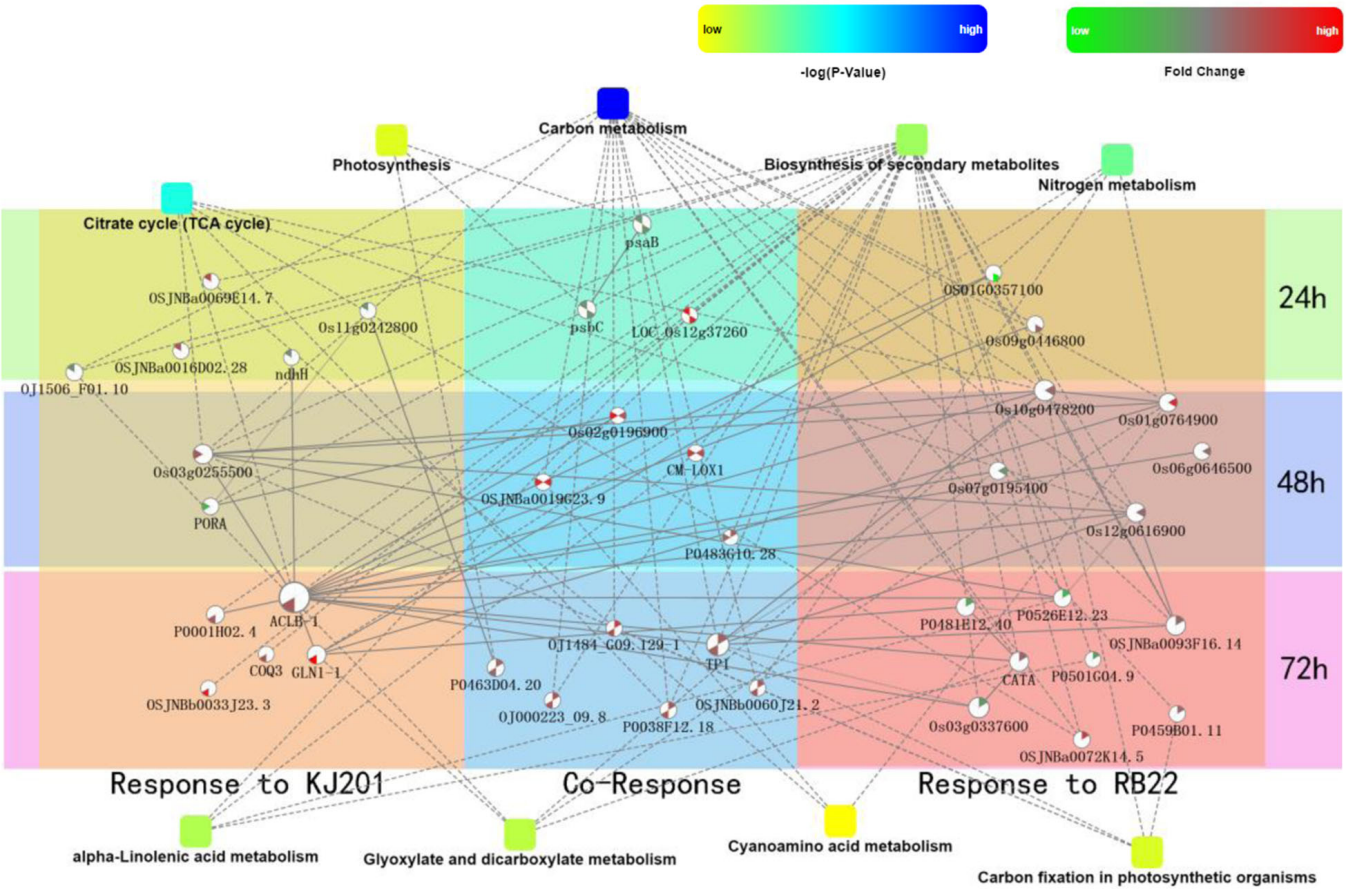
PPI networks of DEPs in NPB-Piz-t in response to *M. oryzae* inoculation. Response to KJ201: DEPs specific to comparison KJ201-Piz-t/Mock-Piz-t; Response to RB22: DEPs specific to comparison RB22-Piz-t/Mock-Piz-t; Co-Response: common DEPs in comparisons KJ201-Piz-t/Mock-Piz-t and RB22-Piz-t/Mock-Piz-t. *P*-values were calculated based on a -log scale. Fold change indicates the expression level of protein in the networks and are indicated in the white circle above each protein name

A Protein–Protein Interaction (PPI) network of the identified DEPs was constructed using STRING (Fig. 6). In NPB-Piz-t in response to KJ201, the DEPs were involved primarily in the biosynthesis of secondary metabolites. Specifically, ATP-citrate synthase beta chain protein 1 (ACLB-1) interacted with 15 proteins involved in secondary metabolite biosynthesis, carbon metabolism, cyanoamino acid metabolism, alpha-linolenic acid metabolism, carbon fixation in photosynthetic organisms, TCA cycle, nitrogen metabolism, and glyoxylate and dicarboxylate metabolism. In NPB-Piz-t in response to RB22, the DEPs were involved mainly in carbon metabolism and the biosynthesis of secondary metabolites. Similarly, the common DEPs in NPB-Piz-t in response to both the two avirulent and virulent isolates, were mainly associated with secondary metabolite biosynthesis, carbon metabolism, and photosynthesis.

**Fig. 7:**
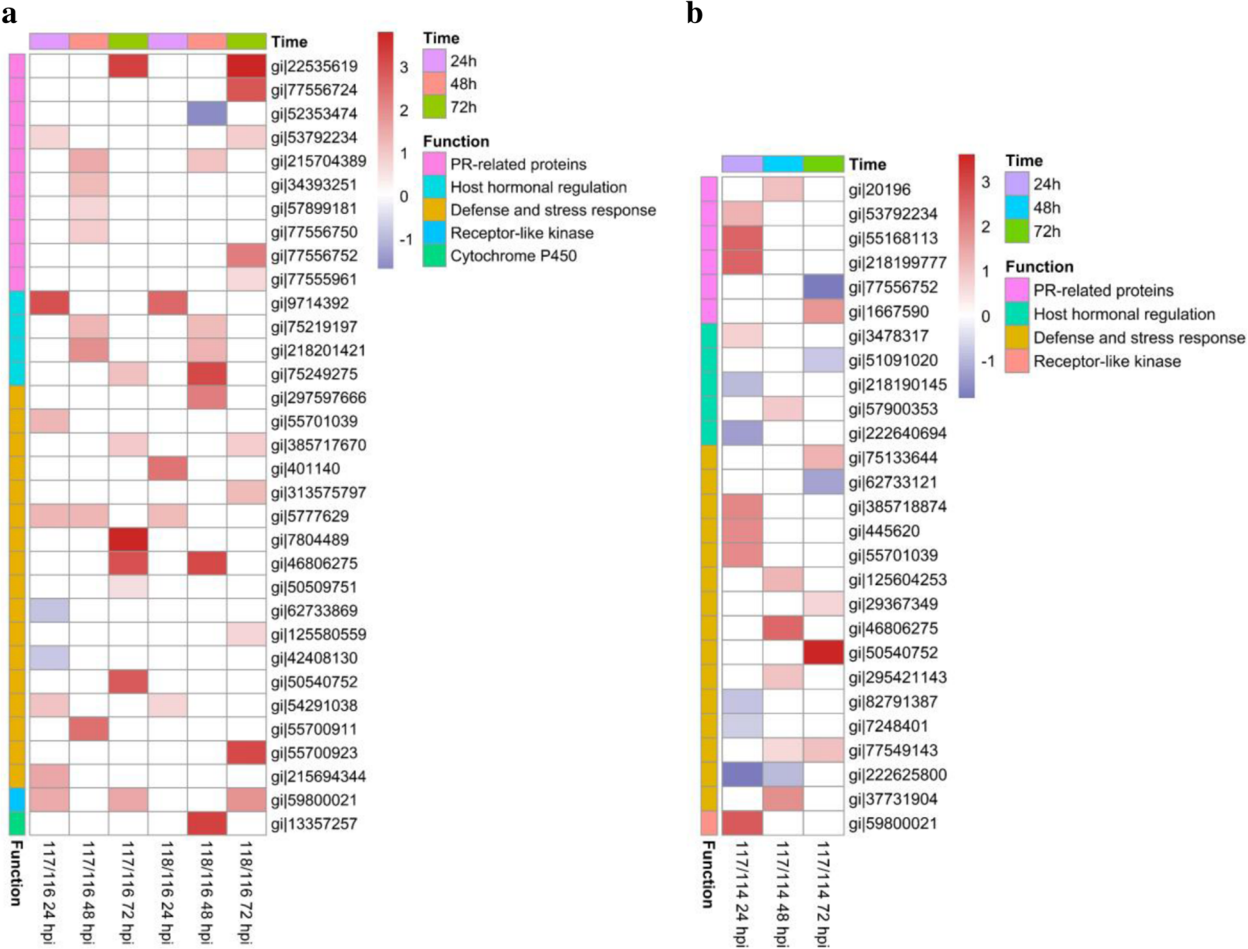
Expression patterns of DEPs may be involved in rice response to pathogens. **a** DEPs in NPB-Piz-t in response to *M. oryzae* inoculation. **b** DEPs in comparison between NPB-Piz-t and NPB in response to *M. oryzae* isolate KJ201. 114: KJ201-inoculated NPB; 116: mock-treated NPB-Piz-t; 117: KJ201-inoculated NPB-Piz-t; 118: RB22-inoculated NPB-Piz-t

Based on GO and BLAST annotations, 33 DEPs that may be involved in rice response to pathogens were identified, including 10 PR proteins, 4 hormonal regulation-related proteins, 17 defense and stress-related proteins, 1 receptor-like kinase, and 1 cytochrome P450 (Fig. 7a, Additional file 3: Table S3). Most of the proteins were upregulated in KJ201-Piz-t or in RB22-Piz-t, except for 1 PR protein gi|52353474 which was downregulated in RB22-Piz-t at 48 hpi, and 2 defense and stress-related proteins, gi|62733869 and gi|42408130, which were downregulated in KJ201-Piz-t at 24 hpi.

### Differential proteomic profiles of NPB and NPB-Piz-t in response to *M. oryzae* isolate KJ201

To obtain a more comprehensive profile of the proteome involved in *Piz-t*-mediated blast resistance, the proteomic profiles of KJ201-Piz-t and KJ201-NPB were compared. In total, 93 proteins were differentially expressed between KJ201-Piz-t and KJ201-NPB (Additional file 4: Table S4).

GO analysis of DEPs in comparison KJ201-Piz-t/KJ201-NPB showed that the most common biological process categories were associated with metabolic and biosynthetic processes; the most common cellular component categories were associated with cytoplasm and cytoplasmic parts; and the most common molecular function categories were related to catalytic activity (Additional file 5: Figure S1a, Additional file 6: Table S5). KEGG analysis revealed that the DEPs functioned mainly in carbon metabolism, metabolic pathways, biosynthesis of secondary metabolites, sulfur metabolism, porphyrin and chlorophyll metabolism, biosynthesis of amino acids, and carbon fixation in photosynthetic organisms (Additional file 5: Figure S1b, Additional file 6: Table S5).

**Fig. 8:**
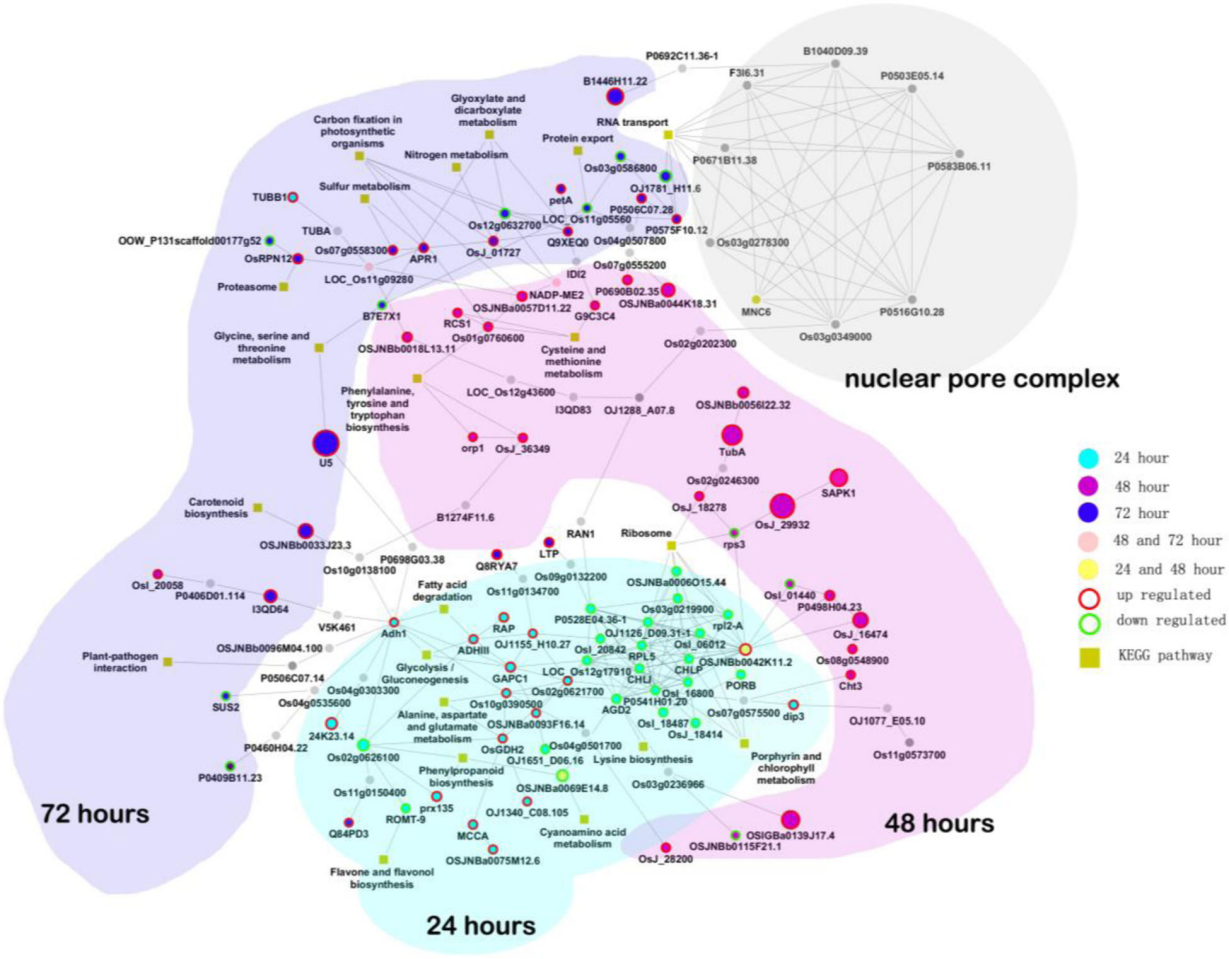
PPI networks of DEPs in comparison between NPB-Piz-t and NPB in response to *M. oryzae* isolate KJ201. 24 h: 24 hpi; 48 h: 48 hpi; 72 h: 72 hpi

PPI analysis revealed a complex network of the DEPs in comparison KJ201-Piz-t/KJ201-NPB (Fig. 8). At 24 hpi, while the proteins upregulated specifically in KJ201-Piz-t were involved primarily in fatty acid degradation; glycolysis/gluconeogenesis; and alanine, aspartate, and glutamate metabolism, a number of downregulated proteins were associated with porphyrin and chlorophyll metabolism and ribosome. At 48 hpi, most of the DEPs were upregulated in KJ201-Piz-t, including proteins associated with cysteine and methionine metabolism, and phenylalanine, tyrosine, and tryptophan biosynthesis. Specifically, OSJNBb0042k11.2 (ribosomal protein L17-like protein) interacted with several downregulated proteins at 24 hpi and with 4 proteins up- or downregulated at 48 hpi. At 72 hpi, DEPs were mainly associated with carbon fixation in photosynthetic organisms, glyoxylate and dicarboxylate metabolism, and sulfur metabolism. It is notable that several proteins related to RNA transport were linked to the nuclear pore complex (by network extension), suggesting that the activation of defense may be associated with gene regulation in the nuclei.

Twenty seven proteins that may be involved in rice response to pathogens were identified from DEPs in comparison KJ201-Piz-t/KJ201-NPB, including 6 PR proteins, 5 hormonal regulation-related proteins, 15 defense and stress-related proteins, and 1 receptor-like kinase (Fig. 7b, Additional file 3: Table S3). PR proteins and the receptor-like kinase were mainly upregulated in KJ201-Piz-t. In contrast, hormonal regulation-related proteins and defense and stress-related proteins displayed differential expression between KJ201-Piz-t and KJ201-NPB.

### Identification of common DEPs in comparisons KJ201-Piz-t/KJ201-NPB and KJ201-Piz-t/RB22-Piz-t

The DEPs common to KJ201-Piz-t/KJ201-NPB and KJ201-Piz-t/RB22-Piz-t were identified. The KJ201-Piz-t interaction was incompatible, whereas the KJ201-NPB and RB22-Pizt interactions were compatible (Fig. 1b). Thus, the DEPs common to both comparisons may be strictly related to *Piz-t*-mediated rice blast resistance. In total, 9 proteins were differentially expressed between KJ201-Piz-t and KJ201-NPB and between KJ201-Piz-t and RB22-Piz-t (Table 2). However, two proteins, gi|57900129 and gi|218188004, showed inconsistent patterns of expression; thus, seven proteins were identified as common DEPs between the two comparisons (Table 2). One of the reasons for low common DEPs in comparisons KJ201-Piz-t/KJ201-NPB and KJ201-Piz-t/RB22-Piz-t may be attributed to the differences of genetic background between *M. oryzae* isolates KJ201 and RB22, and between NPB and NPB-Piz-t, although NPB-Piz-t was generated from NPB background. Among these common DEPs, alcohol dehydrogenase I (gi|34787317) and receptor-like protein kinase (gi|59800021) were upregulated in KJ201-Piz-t as compared with KJ201-NPB or RB22-Piz-t at 24 hpi; endochitinase (gi|20196) was upregulated in KJ201-Piz-t at 48 hpi, and similar to rubisco large subunit (gi|4680202) and hypothetical protein OsJ_24720 (gi|222637272) were upregulated in KJ201-Piz-t at 72 hpi. The two proteins, hypothetical protein OsJ_12578 (gi|222625800) and NADP-dependent malic enzyme (gi|54606800), were downregulated in KJ201-Piz-t as compared with KJ201-NPB or RB22-Piz-t at 48 and 72 hpi, respectively.

**Table 2:** Common DEPs in comparisons KJ201-Piz-t/KJ201-NPB and KJ201-Piz-t/RB22/Piz-t

Accession no.	Protein description	Fold change		Differentially expression pattern	
		KJ201-Piz-t/KJ201-NPB	KJ201-Piz-t/RB22/Piz-t	KJ201-Piz-t/KJ201-NPB	KJ201-Piz-t/RB22/Piz-t
gi|34787317	Alcohol dehydrogenase I [*Oryza rufipogon*]	1.690	1.871	Up at 24 hpi	Up at 24 hpi
gi|59800021	Receptor-like protein kinase [*Oryza sativa* Japonica Group]	6.607	4.875	Up at 24 hpi	Up at 24 hpi
gi|20196	Endochitinase, OsCHIT7 [*Oryza sativa*]	2.089	4.920	Up at 48 hpi	Up at 48 hpi
gi|4680202	Similar to rubisco large subunit [*Sorghum bicolor*]	1.514	3.404	Up at 72 hpi	Up at 72 hpi
gi|222637272	Hypothetical protein OsJ_24720 [*Oryza sativa* Japonica Group]	2.421	3.221	Up at 72 hpi	Up at 72 hpi
gi|222625800	Hypothetical protein OsJ_12578 [*Oryza sativa* Japonica Group]	0.530	0.391	Down at 48 hpi	Down at 48 hpi
gi|54606800	NADP dependent malic enzyme [*Oryza sativa* Japonica Group]	0.637	0.619	Down at 72 hpi	Down at 72 hpi
gi|57900129	Putative transaldolase [*Oryza sativa* Japonica Group]	1.528	0.535	Up at 24 hpi	Down at 72 hpi
gi|218188004	Hypothetical protein OsI_01440 [*Oryza sativa* Indica Group]	0.366	1.675	Down at 48 hpi	Up at 48 hpi

## Discussion

We used iTRAQ to investigate proteome expression profiles during compatible and incompatible interactions of rice NPB and NPB-Piz-t lines with *M. oryzae* isolates. Our analysis identified a number of DEPs that may be involved in rice-*M. oryzae* interactions and revealed common DEPs in NPB-Piz-t during the resistance reaction to *M. oryzae*.

### Carbohydrate metabolism and energy production in response to *M. oryzae* infection

Photosynthesis is one of the most fundamental physiological processes in plants. However, the photosystem is vulnerable to damage by plant pathogens, and if not repaired in time, it loses its function (Che et al. 2013). Reducing the photosynthetic rate to allocate resources in defense against pathogens which compromise photosynthesis has been suggested as an effective defense mechanism in the early infection stages (Li et al. 2015). Hanssen et al. (2011) showed that a number of photosynthesis-related genes were downregulated in tomato plants infected with *Pepino mosaic virus* during early stages of infection. Similarly, comparative phosphoproteomic analysis revealed that a number of photosynthesis-related phosphoproteins were downregulated in rice in both compatible and incompatible interactions with *M. oryzae* (Li et al. 2015). In addition, our research showed that two important photosynthesis-related proteins, psaB (gi|42795618) and psbC (gi|68565738) (Fig. 6, Additional file 1: Table S1) were downregulated in both compatible and incompatible interactions at 24 hpi. In contrast, at 48 or 72 hpi, a photosynthesis-related protein, P0463D04.20 (gi|52077301), and several proteins related to carbon fixation in photosynthesis, including Os03g0255500 (gi|291048382), OJ1484_G09.129–1 (gi|257665951), OSJNBb0060J21.2 (gi|81686700), TPI (gi|385717670), Os10g0478200 (gi|75141370), and P0459B01.11 (gi|41052955), were found to be upregulated in both compatible and incompatible interactions (Fig. 6, Additional file 1: Table S1). The respiratory pathway of the TCA cycle is essential for energy production (Fernie et al. 2004). In the present study, six TCA cycle-related proteins, Os03g0255500 (gi|291048382), Os12g0616900 (gi|77557068), Os10g0478200 (gi|68565738), ACLB-1 (gi|75249275), P0038F12.18 (gi|81686717), and OSJNBa0072K14.5 (gi|21740743), were found to be upregulated in compatible or incompatible interactions at 48 or 72 hpi (Fig. 6, Additional file 1: Table S1). The upregulation of photosynthesis-and TCA cycle-related proteins at late infection stages suggests that restoration of the photosynthetic system in rice promotes recovery from *M. oryzae* infection.

### Defense-related proteins involved in NPB and NPB-Piz-t responses to *M. oryzae* infection

Plant PR proteins play an important role in defenses against pathogens (van Loon et al. 2006). In this study, 12 PR proteins were identified as DEPs between the *M. oryzae*-inoculated NPB and/or NPB-Piz-t and the mock-treated rice leaves samples (Fig. 7, Additional file 3: Table S3). While OsOC-2 (gi|52353474) exhibited less expression levels in KJ201-NPB (compared with Mock-NPB, at 48 hpi) and in RB22-Piz-t (compared with Mock-Pizt, at 48 hpi), respectively, the rest 11 PRs were expressed higher in the *M. oryzae*-inoculated samples than that in the mock-treated samples. In comparisons KJ201-Piz-t/KJ201-NPB and KJ201-Piz-t/RB22-Piz-t, 7 PRs were identified as DEPs (Fig. 7, Additional file 3: Table S3). Interestingly, compared with KJ201-NPB or RB22-Piz-t, 5 out of 7 PRs, OsCHIT7/endochitinase (gi|20196), BBTI-4/RBBI3–1 (gi|53792234), OsCHIB1 (gi|55168113), Gns12 (gi|218199777), and OsLTP2 (gi|1667590) were identified to be differentially upregulated in KJ201-Piz-t. The results suggest that some PRs may be involved more specifically in Piz-t-mediated resistance response.

The receptor-like kinases (RLKs) are key regulators of plant development and defense (Gao and Xue 2012). The rice genome contains more than 1131 RLKs that are involved in regulating various physiological processes, including defense and disease resistance (Shiu et al. 2004). In this study, a receptor-like kinase protein (gi|59800021) was found to be differentially upregulated in *M. oryzae*-inoculated NPB and NPB-Piz-t at 24 hpi or 72 hpi, compared with the mock-treated NPB or NPB-Piz-t samples (Additional file 7: Table S6). gi|59800021 was also upregulated in KJ201-Piz-t when compared with KJ201-NPB or RB22-Piz-t (Additional file 7: Table S6). gi|59800021 contains two salt stress response/antifungal domains. We speculate that this receptor-like kinase protein would be worthy of functional investigation as it may play an important role in rice defenses against blast fungus.

Recent studies have revealed that AvrPiz-t function to suppress host basal defense (Li et al. 2009; Park et al. 2012). By Y2H screening, 12 APIPs have been identified (Park et al. 2012), and APIP5 (Wang et al. 2016), APIP6 (Park et al. 2012), APIP7 (Shi et al. 2018), APIP10 (Park et al. 2016), and APIP12 (Tang et al. 2017) have been functionally confirmed as targets of AvrPiz-t. Interestingly, in this study, we observed that a putative bowman birk trypsin inhibitor (gi|53792234) was induced to higher levels in KJ201-NPB than that in Mock-NPB at 72 hpi, in KJ201-Piz-t than that in Mock-Piz-t at 24 hpi, in RB22-Piz-t than that in Mock-Piz-t at 72 hpi, and in KJ201-Piz-t than that in KJ201-NPB at 24 hpi (Additional file 7: Table S6). The bowman birk trypsin inhibitor was previously identified as APIP4 by Y2H (Park et al. 2012). Taken together, the APIP4/bowman birk trypsin inhibitor might be involved in AvrPiz-t/Piz-t networks.

## Conclusions

In this study, comparative proteome profiling of the transgenic NPB-Piz-t and wild type NPB inoculated with an avirulent *M. oryzae* isolate KJ201 and a virulent isolate RB22 at 24, 48, and 72 hpi were investigated using iTRAQ analysis. A total of 114 and 118 DEPs were identified in comparisons KJ201-Piz-t/Mock-Pizt and RB22-Piz-t/Mock-Pizt, respectively. A number of DEPs that may be involved in rice response to pathogens were identified, including PR proteins, hormonal regulation-related proteins, defense and stress response-related proteins, receptor-like kinase, and cytochrome P450. There were 7 common DEPs between the comparisons KJ201-Piz-t/RB22-Piz-t and KJ201-Piz-t/KJ201-NPB, including alcohol dehydrogenase I, receptor-like protein kinase, endochitinase, similar to rubisco large subunit, NADP-dependent malic enzyme, and two hypothetical proteins. These results provide a valuable resource for studying the mechanism of *Piz-t*-mediated defense in rice.

## Methods

### Plant materials, fungal isolates, and *M. oryzae* inoculation

The rice cultivar Nipponbare (NPB), the transgenic Nipponbare line harboring the *Piz-t* gene (NPB-Piz-t) (Park et al. 2016), and the *M. oryzae* isolates KJ201 (avirulent to *Piz-t*) and RB22 (virulent to *Piz-t*) were used in this study. *M. oryzae* inoculations were performed in a greenhouse following a previously described procedure (Tian et al. 2016). Rice seedlings were grown in the greenhouse for approximately 2 weeks and were spray-inoculated with spores at a concentration of 5 × 10^5^ spores mL^− 1^. The inoculated seedlings were maintained under high humidity, and leaves were collected at 24, 48, and 72 hpi, frozen in liquid nitrogen, and stored at − 80 °C until protein extraction.

### Protein extraction, digestion, and iTRAQ labeling

Total protein was extracted from leaves of control and inoculated plants according to previously described procedures (Yang et al. 2013). The proteins were reduced using dithiothreitol and alkylated using iodoacetamide prior to trypsin digestion. The digested proteins were labeled using an iTRAQ Reagents 8PLEXKit (Applied Biosystems, FosterCity, CA, USA) according to the manufacturer’s instructions. The samples of mock-treated NPB and NPB-Piz-t were labeled with iTRAQ tags 113 and 116; KJ201-inoculated NPB and NPB-Piz-t samples were labeled with iTRAQ tags 114 and 117; and RB22-inoculated NPB and NPB-Piz-t were labeled with iTRAQ tags 115 and 118, respectively (Fig. 1a). The samples of NPB and NPB-Piz-tat 24, 48, and 72 hpi were run in three independent experiments.

### 2D high-performance liquid chromatography (HPLC) separation, tandem mass spectrometry (MS/MS) analysis, protein identification, and relative quantification

Strong cation exchange, reversed-phase nanoflow HPLC, and MS/MS measurements were performed as described previously (Yang et al. 2016). The MS/MS data were processed with ProteinPilot software (Applied Biosystems) against a local rice protein database from the National Center for Biotechnology Information (NCBI) with parameter settings as described (Yang et al. 2016). Protein identification was based on a FDR of 1%, and proteins with a fold change value of ≥1.5 or ≤ 0.67 and a *P*-value of < 0.05 were regarded as significantly differentially expressed.

### Bioinformatics analysis

Biological processes, cellular components, and molecular functions of DEPs were determined by GO database annotation (http://www.geneontology.org/). Protein signaling pathways were elucidated using the Kyoto Encyclopedia of Genes and Genomes (KEGG) database(http://www.genome.jp/kegg/pathway.html). Pathways enriched at *P*-value < 0.05 were considered significant. Protein–protein interaction (PPI) analysis was performed using STRING (www.string-db.org).

### qRT-PCR

Total RNA was extracted from rice leaves using Trizol reagent (Invitrogen, Carlsbad, CA, USA) according to the manufacturer’s protocol, followed by DNase I (Takara, Dalian, China) treatment. First-strand cDNA was generated using a RevertAid First Strand cDNA Synthesis Kit (Thermo Scientific, Waltham, MA, USA). qRT-PCR was performed on an ABI Prism 7500 Detection System (Applied Biosystems) using a SYBR Green Real-time PCR Master Mix (Takara). The primers used for qRT-PCR are listed in Additional file 8: Table S7.

### Western blot analysis

Total protein was extracted from rice leaves using an extraction buffer (50 mM Tris-MES, pH 8.0, 0.5 M Suc, 1 mMMgCl2, 10 mM EDTA, 5 mM DTT, 100 μM MG132), and plant protease inhibitor cocktail as described (Park et al. 2012). The protein concentration was measured using a Bio-Rad protein assay kit (Bio-Rad, Hercules, CA). Proteins (30 μg) were separated using 10% SDS-PAGE and then electrotransferred onto a polyvinylidene fluoride membrane (Millipore, Billerica, MA, USA). Immunoblotting was conducted using standard protocols. Anti-OsGH1, anti-OsGH18, and anti-OsCHIT7 (Beijing Protein Innovation, Beijing, China) were used at a dilution of 1:2000. Horseradish peroxidase-conjugated goat anti-rabbit antibody (Jackson ImmunoResearch, West Grove, PA, USA) was used at a dilution of 1:10,000. Chemiluminescence was detected using Pierce ECL substrate (Thermo Scientific) followed by exposure to hyperfilm.

## Additional files

Additional file 1: **Table S1.** DEPs in comparisons KJ201-NPB/Mock-NPB, RB22-NPB/Mock-NPB, KJ201-Piz-t/Mock-Piz-t, and RB22-Piz-t//Mock-Piz-t. (XLS 152 kb)

Additional file 2: **Table S2.** GO and KEGG analyses of DEPs in comparisons KJ201-Piz-t/Mock-Piz-t, and RB22-Piz-t//Mock-Piz-t. (XLSX 84 kb)

Additional file 3: **Table S3.** Identification of DEPs that may be involved in rice response to pathogens. (DOCX 27 kb)

Additional file 4: **Table S4.** DEPs in comparison KJ201-Piz-t/KJ201-NPB. (XLSX 23 kb)

Additional file 5: **Figure S1.** GO (a) and KEGG (b) analyses of DEPs in comparison between NPB-Piz-t and NPB in response to *M. oryzae* isolate KJ201. (PPTX 434 kb)

Additional file 6: **Table S5.** GO and KEGG analyses of DEPs in comparison KJ201-Piz-t/KJ201-NPB. (XLSX 47 kb)

Additional file 7: **Table S6.** Differentially expression pattern of receptor-like protein kinase (gi|59800021) and putative bowman birk trypsin inhibitor (gi|53792234). (DOCX 16 kb)

Additional file 8: **Table S7.** Primers used for qRT-PCR analysis in this study. (DOC 30 kb)

## Abbreviations

APIPs: AvrPiz-t interacting proteins
DEPs: Differentially expressed proteins
FDR: False discovery rate
GO: Gene ontology
hpi: Hours post-inoculation
HPLC: High-performance liquid chromatography
iTRAQ: Isobaric tags for relative and absolute quantification
KEGG: Kyoto encyclopedia of genes and genomes
PPI: Protein–protein interaction
PR: Pathogenesis-related
qRT-PCR: Quantitative reverse-transcription PCR
