# Supplementary material for: Proteomic analysis of the defense response to Magnaporthe oryzae in rice harboring the blast resistance gene Piz-t
Source: Rice (N Y). 2018 Aug 15;11:47. doi: 10.1186/s12284-018-0240-3 (PMC6093832; doi:10.1186/s12284-018-0240-3)
Supplement: Supplementary file 3 — Table S3. Identification of DEPs that may be involved in rice response to pathogens. (DOCX 27 kb) [file 12284_2018_240_MOESM3_ESM.docx]

**Additional file 3: Table S3.** Identification of DEPs that may be involved in rice response to pathogens

| Term/Gene Family | Accession no. | Gene name | Differentially expression pattern | | | | | |
| --- | --- | --- | --- | --- | --- | --- | --- | --- |
|  |  |  | KJ201-NPB/Mock-NPB | RB22-NPB/Mock-NPB | KJ201-Piz-t/Mock-Piz-t | RB22-Piz-t/Mock-Pizt | KJ201-Piz-t/KJ201-NPB | KJ201-Piz-t/RB22-Piz-t |
| PR-related proteins | | | | | | | | |
| PR1 | gi\|22535619 | OsPR1#011/PR-1b | ̶ | ̶ | 9.91 (72 hpi) | 13.68 (72 hpi) | ̶ | ̶ |
| PR2 | gi\|4884530 | Gns10 | 4.02 (48 hpi) | ̶ | ̶ | ̶ | ̶ | ̶ |
| PR3 | gi\|20196 | OsCHIT7 | ̶ | ̶ | ̶ | ̶ | 2.09 (48 hpi) | 4.92 (48 hpi) |
| PR5 | gi\|77556724 | TLP-D34 | 14.45 (72 hpi) | 9.55 (72 hpi) | ̶ | 7.59 (72 hpi) | ̶ | ̶ |
| PR6 | gi\|52353474 | OsOC-2 | 0.30 (48 hpi) | ̶ | ̶ | 0.31 (48 hpi) | ̶ | ̶ |
|  | gi\|53792234 | BBTI-4/RBBI3-1 | 3.08 (72 hpi) | ̶ | 1.66 (24 hpi) | 1.92 (72 hpi) | 2.40 (24 hpi) | ̶ |
| PR8 | gi\|55168113 | OsCHIB1 | ̶ | ̶ | ̶ | ̶ | 6.03 (24 hpi) | ̶ |
|  | gi\|215704389 | Gns12 | ̶ | ̶ | 2.83 (48 hpi) | 2.11 (48 hpi) | ̶ | ̶ |
|  | gi\|218199777 | Gns12 | ̶ | ̶ | ̶ | ̶ | 6.03 (24 hpi) | ̶ |
| PR9 | gi\|34393251 | POX22.3 | 5.75 (48 hpi) | 2.78 (48 hpi) | 2.36 (48 hpi) | ̶ | ̶ | ̶ |
|  | gi\|57899181 | POX22.3 | ̶ | ̶ | 1.67 (48 hpi) | ̶ | ̶ | ̶ |
| PR10 | gi\|77556750 | RSOsPR10 | ̶ | ̶ | 1.91 (48 hpi) | ̶ | ̶ | ̶ |
|  | gi\|77556752 | PR-10b | 15.42 (72 hpi) | 8.63 (72 hpi) | ̶ | 4.79 (72 hpi) | 0.28 (72 hpi) | ̶ |
|  | gi\|77556755 | PR-10a/PBZ1 | 7.31 (72 hpi) | 5.97 (72 hpi) | ̶ | ̶ | ̶ | ̶ |
| PR14 | gi\|1667590 | OsLTP2 | ̶ | ̶ | ̶ | ̶ | 3.22 (72 hpi) | ̶ |
| PR16 | gi\|77555961 | OsGLP8-12 | ̶ | ̶ | ̶ | 1.64 (72 hpi) | ̶ | 0.53 (48 hpi) |

**Additional file 3: Table S3.** Identification of DEPs that may be involved in rice response to pathogens (continued)

| Host hormonal regulation | | | | | | | | |
| --- | --- | --- | --- | --- | --- | --- | --- | --- |
| SA | gi\|3478317 | ADH1 | ̶ | ̶ | ̶ | ̶ | 1.69 (24 hpi) | ̶ |
|  | gi\|51091020 | SUS2 | 2.13 (72 hpi) | ̶ | ̶ | ̶ | 0.62 (72 hpi) | ̶ |
|  | gi\|218190145 | HEMC | ̶ | ̶ | ̶ | ̶ | 0.53 (24 hpi) | ̶ |
| JA | gi\|9714392 | LOC_Os12g37260 | ̶ | ̶ | 8.32 (24 hpi) | 6.31 (24 hpi) | ̶ | ̶ |
|  | gi\|75219197 | CBSX1 | ̶ | ̶ | 2.44 (48 hpi) | 2.31 (48 hpi) | ̶ | ̶ |
|  | gi\|218201421 | OsI_29815 | 9.64 (48 hpi) | ̶ | 3.91 (48 hpi) | 2.61 (48 hpi) | ̶ | ̶ |
| ET | gi\|57900353 | ASP3 | ̶ | ̶ | ̶ | ̶ | 1.92 (48 hpi) | ̶ |
|  | gi\|222640694 | DAP | ̶ | ̶ | ̶ | ̶ | 0.41 (24 hpi) | ̶ |
| BR | gi\|75249275 |  | 1.92 (24 hpi) | ̶ | 2.15 (72 hpi) | 9.12 (48 hpi) | ̶ | ̶ |
| Defense and stress response | | | | | | | | |
| Defense to stress | gi\|75133644 | Hsp90-3 | ̶ | ̶ | ̶ | ̶ | 2.49 (72 hpi) | ̶ |
| Defense to virus | gi\|62733121 | P37107 | ̶ | ̶ | ̶ | ̶ | 0.43 (72 hpi) | ̶ |
| Innate immune response | gi\|297597666 | Os01g0764900 | ̶ | ̶ | ̶ | 4.92 (48 hpi) | ̶ | 0.22 (48 hpi) |
|  | gi\|385718874 | RBG7 | ̶ | ̶ | ̶ | ̶ | 4.02 (24 hpi) | ̶ |
| Peroxidase/  Peroxidase precursor | gi\|445620 | PER52 | ̶ | ̶ | ̶ | ̶ | 3.73 (24hpi) | ̶ |
|  | gi\|55700899 | B1135C02 | 2.17 (72 hpi) | 2.09 (72 hpi) | ̶ | ̶ | ̶ | ̶ |
|  | gi\|55700927 | OSJNBa0082C09 | 8.79 (72 hpi) | ̶ | ̶ | ̶ | ̶ | ̶ |
|  | gi\|55701039 | PER86 | 3.28 (72 hpi) | 2.19 (72 hpi) | 2.47 (24 hpi) | ̶ | 3.87 (24 hpi) | ̶ |
|  | gi\|55701117 | OSJNBa0015O22 | 1.69 (72 hpi) | ̶ | ̶ | ̶ | ̶ | ̶ |

**Additional file 3: Table S3.** Identification of DEPs that may be involved in rice response to pathogens (continued)

| Response to abiotic stimulus | gi\|385717670 | TPI | ̶ | ̶ | 1.94 (72 hpi) | 1.85 (72 hpi) | ̶ | ̶ |
| --- | --- | --- | --- | --- | --- | --- | --- | --- |
| Response to bacterium | gi\|125604253 | GSTF10 | ̶ | ̶ | ̶ | ̶ | 2.31 (48 hpi) | ̶ |
| Response to biotic stimulus | gi\|401140 | SUS1 | 2.54 (24 hpi) | 0.44 (24 hpi) | ̶ | 5.40 (24 hpi) | ̶ | ̶ |
|  | gi\|313575797 | Os03g0276500 | ̶ | 0.43 (48 hpi) | ̶ | 2.31 (72 hpi) | ̶ | ̶ |
| Response to chitin | gi\|29367349 | Q9MA63 | ̶ | ̶ | ̶ | ̶ | 1.60 (72 hpi) | ̶ |
| Response to stimulus | gi\|5777629 | OSJNBa0039K24.4 | ̶ | ̶ | 2.47 (24 hpi)，2.42（48 hpi） | 2.29 (24 hpi) | ̶ | ̶ |
|  | gi\|7804489 | OS02G0735200 | ̶ | ̶ | 14.06 (72 hpi) | ̶ | ̶ | ̶ |
|  | gi\|46806275 | OSJNBb0056I22.32 | ̶ | 8.02 (48 hpi) | 9.12 (48 hpi) | ̶ | 5.60 (48 hpi) | ̶ |
|  | gi\|50509751 | OSJNBa0050F10.39-2 | ̶ | ̶ | 1.53 (72 hpi) | ̶ | ̶ | ̶ |
|  | gi\|62733869 | Os11g0242800 | ̶ | 0.48 (72 hpi) | 0.58 (24 hpi) | ̶ | ̶ | ̶ |
|  | gi\|125580559 | CATA | ̶ | ̶ | ̶ | 1.66 (72 hpi) | ̶ | ̶ |
| Response to stress | gi\|42408130 | OJ1506_F01.10 | ̶ | ̶ | 0.61 (24 hpi) | ̶ | ̶ | ̶ |
|  | gi\|50540752 | OSJNBb0033J23.3 | ̶ | ̶ | 7.24 (72 hpi) | ̶ | 12.25 (72 hpi) | ̶ |
|  | gi\|54291038 | P0481E08.35 | 1.59 (72 hpi) | ̶ | 2.05 (24hpi) | 1.69 (24 hpi) | ̶ | ̶ |
|  | gi\|55700911 | P0483G10.32 | ̶ | ̶ | 5.55 (48 hpi) | ̶ | ̶ | ̶ |
|  | gi\|55700923 | OSJNBa0082C09.19 | 0.20 (72 hpi) | ̶ | ̶ | 8.79 (72 hpi) | ̶ | 0.10 (72 hpi) |
| Response to wounding | gi\|295421143 | OASA1 | ̶ | ̶ | ̶ | ̶ | 2.01 (48 hpi) | ̶ |
|  | gi\|82791387 | OMT1 | 1.72 (24 hpi) | ̶ | ̶ | ̶ | 0.59 (24 hpi) | ̶ |
| SAR | gi\|7248401 | CPN60B1 | ̶ | 0.55 (24 hpi) | ̶ | ̶ | 0.63 (24 hpi) | ̶ |
|  | gi\|77549143 | PDIL1-1 | 1.99 (48 hpi) | ̶ | ̶ | ̶ | 1.85 (48 hpi), 2.11 (72 hpi) | ̶ |

**Additional file 3: Table S3.** Identification of DEPs that may be involved in rice response to pathogens (continued)

| SAR | gi\|215694344 | OSJNBa0069E14.7 | ̶ | ̶ | 3.08 (24 hpi) | ̶ | ̶ | ̶ |
| --- | --- | --- | --- | --- | --- | --- | --- | --- |
|  | gi\|222625800 | AT5G20950 | ̶ | ̶ | ̶ | ̶ | 0.28 (24 hpi), 0.53 (48 hpi) | 0.39（48 hpi） |
| Stress responsive | gi\|37731904 | At2g31670 | ̶ | ̶ | ̶ | ̶ | 3.60 (48 hpi) | - |
| Receptor-like kinase |  |  |  |  |  |  |  |  |
|  | gi\|59800021 | Receptor-like kinase | 3.70 (72 hpi) | 3.22 (72 hpi) | 2.83 (24 hpi), 3.02 (72hpi) | 3.73 (72 hpi) | 6.61 (24 hpi) | 4.88 (24 hpi) |
| Cytochrome P450 |  |  |  |  |  |  |  |  |
|  | gi\|13357257 | OSJNBa0001O14.16 | ̶ | ̶ | ̶ | 10.00 (48 hpi) | ̶ | 0.23 (48 hpi) |
