# Supplementary material for: Proteomic analysis of the defense response to Magnaporthe oryzae in rice harboring the blast resistance gene Piz-t
Source: Rice (N Y). 2018 Aug 15;11:47. doi: 10.1186/s12284-018-0240-3 (PMC6093832; doi:10.1186/s12284-018-0240-3)
Supplement: Supplementary file 5 — Figure S1. GO (a) and KEGG (b) analyses of DEPs in comparison between NPB-Piz-t and NPB in response to M. oryzae isolate KJ201. (PPTX 434 kb) [file 12284_2018_240_MOESM5_ESM.pptx]

## Slide 1
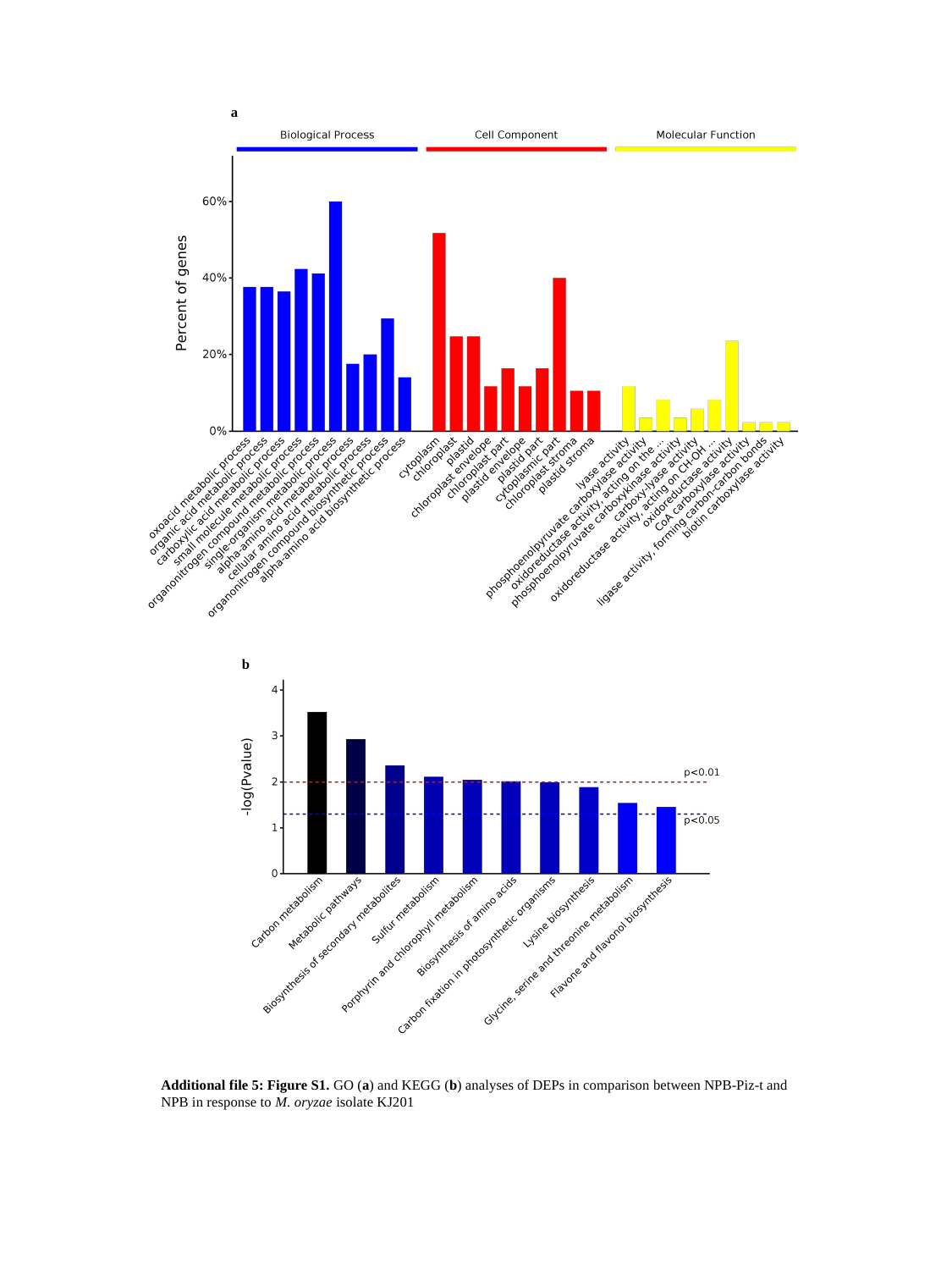

a
b
Additional file 5: Figure S1. GO (a) and KEGG (b) analyses of DEPs in comparison between NPB-Piz-t and NPB in response to M. oryzae isolate KJ201
