# Supplementary material for: Proteomic analysis of the defense response to Magnaporthe oryzae in rice harboring the blast resistance gene Piz-t
Source: Rice (N Y). 2018 Aug 15;11:47. doi: 10.1186/s12284-018-0240-3 (PMC6093832; doi:10.1186/s12284-018-0240-3)
Supplement: Supplementary file 7 — Table S6. Differentially expression pattern of receptor-like protein kinase (gi|59800021) and putative bowman birk trypsin inhibitor (gi|53792234). (DOCX 16 kb) [file 12284_2018_240_MOESM7_ESM.docx]

**Additional file 7: Table S6.** Differentially expression pattern of receptor-like protein kinase (gi|59800021) and putative bowman birk trypsin inhibitor (gi|53792234)

| Accession no. | Description | Differentially expression pattern | | | | | | |
| --- | --- | --- | --- | --- | --- | --- | --- | --- |
|  |  | hpi | KJ201-NPB/  Mock-NPB | RB22-NPB/  Mock-NPB | KJ201-Piz-t/  Mock-Piz-t | RB22-Piz-t/  Mock-Pizt | KJ201-Piz-t/  KJ201-NPB | KJ201-Piz-t/  RB22-Piz-t |
| gi\|59800021 | Receptor-like kinase protein | 24 | ̶ | ̶ | 2.831 | ̶ | 6.607 | 4.875 |
|  |  | 72 | 3.698 | 3.221 | 3.020 | 3.733 | ̶ | ̶ |
| gi\|53792234 | Putative bowman birk trypsin inhibitor | 24 | ̶ | ̶ | 1.660 | ̶ | 2.399 | ̶ |
|  |  | 72 | 3.076 | ̶ | ̶ | 1.923 | ̶ | ̶ |
