# Supplementary material for: Proteomic analysis of the defense response to Magnaporthe oryzae in rice harboring the blast resistance gene Piz-t
Source: Rice (N Y). 2018 Aug 15;11:47. doi: 10.1186/s12284-018-0240-3 (PMC6093832; doi:10.1186/s12284-018-0240-3)
Supplement: Supplementary file 8 — Table S7. Primers used for qRT-PCR analysis in this study. (DOC 30 kb) [file 12284_2018_240_MOESM8_ESM.doc]

**Additional file 8: Table S7. Primers used for qRT-PCR analysis in this study**

| Gene Name | Forward primer (5' - 3') | Reverse primer(5' - 3') |
| --- | --- | --- |
| *OsGH18* (gi|55168113) | CCAGGAGACCAGGCTCATTACCA | CAGTTCATCAACAAGCTGCCCAAC |
| *OsGH1* (gi|218199777) | AATGTGGGCATCCCTCTGG | GCTTGGGCGTTGGCTTTT |
| *OsCHIT7* (gi|20196) | GCCGTCGTGCCACGCGGTGAT | TCGTCGGCGGACGGCGTCCACT |
| *Ubiquitin* | AACCAGCTGAGGCCCAAGA | ACGATTGATTTAACCAGTCCATGA |
